# Active and Inactive Tuberculosis Classification Using Convolutional Neural Networks with MLP-Mixer

**DOI:** 10.3390/bioengineering12060630

**Published:** 2025-06-09

**Authors:** Beanbonyka Rim, Hyeonung Jang, Hongchang Lee, Wangsu Jeon

**Affiliations:** 1 Department of Software Convergence, Soonchunhyang University, Asan 31538, Republic of Korea; rim.beanbonyka@sch.ac.kr; 2 Haewootech Co., Ltd., Busan 46742, Republic of Korea; hwjang@haewootech.co.kr (H.J.); hclee@haewootech.co.kr (H.L.); 3 Department Computer Engineering, Kyungnam University, Changwon 51767, Republic of Korea

**Keywords:** active tuberculosis, CNN, computer-aided diagnosis system, deep learning, EfficientNet, inactive tuberculosis, latent TB screening, lung disease detection, MLP-Mixer

## Abstract

Early detection of tuberculosis plays a critical role in effective treatment management. Like active tuberculosis, early identification of inactive forms such as latent or healed tuberculosis is essential to prevent future reactivation. In this study, we developed a deep-learning-based binary classification model to distinguish between active and inactive tuberculosis cases. Our model architecture incorporated an EfficientNet backbone with an MLP-Mixer classification head and was fine-tuned on a dataset annotated by Cheonan Soonchunhyang Hospital. To enhance predictive performance, we applied transfer learning using weights pre-trained on the JFT-300M dataset via the Noisy Student training method. Unlike conventional models, our approach achieved competitive results, with an accuracy of 96.3%, a sensitivity of 95.9%, and a specificity of 96.6% on the test set. These promising outcomes suggest that our model could serve as a valuable asset to support clinical decision-making and streamline early screening workflows for latent tuberculosis.

## 1. Introduction

Tuberculosis (TB) remains a major public health challenge in South-East Asia, accounting for nearly 45% of global TB cases and approximately half of all TB-related deaths, according to the WHO South-East Asia region [1]. In response to this burden, the World Health Organization (WHO) launched the End TB Strategy, aiming to reduce TB-related mortality by 95% and TB incidence by 90% by the year 2035 [2]. Several studies have identified gaps in diagnosis and treatment linkage as key contributors to the persistent TB burden [3]. As a result, early TB screening is considered a critical step toward improving diagnosis rates and the timely initiation of treatment.

The global TB diagnostics market was valued at USD 1.71 billion in 2021 and is projected to reach USD 3.01 billion by 2030, reflecting a compound annual growth rate (CAGR) of 6.5% over the forecast period [4]. The Asia–Pacific region is expected to experience the fastest growth during this period. In response, both public and private sectors are increasingly collaborating to raise awareness of TB infections and promote participation in screening programs. Consequently, affordable and accessible TB screening has become a critical priority across the Asia–Pacific region to enhance diagnosis and treatment engagement.

Differentiating latent TB from normal cases is essential as individuals with latent TB are infected with Mycobacterium TB but do not exhibit signs of active disease. TB bacteria can persist in the body for years without causing symptoms. Individuals with latent infection do not feel ill and cannot transmit the bacteria to others. However, if the bacteria become active, latent TB can progress into active TB disease [5]. Early screening and timely treatment of latent TB infection are critical as they significantly reduce the risk of progression to active TB.

In 2019, the prevalence of latent TB infection was reported to be as high as 43.75% in Vietnam [6]. It is projected that latent TB infection could lead to active TB cases at a rate of 16.5 and 8.3 per 100,000 population by the years 2035 and 2050, respectively [7]. As such, early detection of latent TB plays a crucial role in ensuring timely access to preventive TB therapies.

Diagnostic tests commonly used to detect TB infection include (1) the Mantoux tuberculin skin test; (2) interferon gamma release assays (IGRAs); and (3) chest X-rays (CXRs) [8]. Among these, chest X-rays are the most widely used due to their cost-effectiveness. However, because CXRs are two-dimensional grayscale representations of three-dimensional anatomical structures, they often exhibit high sensitivity but limited specificity [8]. Despite these limitations, CXR imaging remains the primary modality for TB screening, supported by advantages such as low radiation exposure, portability, and integration with Picture Archiving and Communication Systems (PACSs) [9].

The rapid advancement of Artificial Intelligence (AI) in medical imaging over the past decade has demonstrated significant improvements in performance [10,11]. Convolutional neural networks (CNNs) have led image classification tasks due to their ability to capture spatial hierarchies through localized feature extraction. Recently, Transformer-based models [12] have gained attention for their capacity to model long-range dependencies and global context via self-attention mechanisms. However, their application in medical imaging faces notable limitations. Transformers typically demand much larger datasets and greater computational resources to achieve robust performance, conditions that are often impractical in clinical environments where annotated datasets are limited. Moreover, Transformers lack inherent inductive biases for spatial relationships and local feature interactions strengths that CNNs naturally possess through convolutional operations. These properties allow CNNs to generalize efficiently from smaller datasets, a critical advantage in healthcare where annotated data are often scarce.

In addition, CNNs support precise localization of pathological features through established visualization techniques such as Grad-CAM and heatmaps [13,14,15], providing interpretable insights crucial for clinical decision-making. In contrast, visual explanations generated by Transformer-based models tend to be less intuitive and more computationally intensive, reducing their practical value in clinical workflows. Given these considerations, CNNs remain better suited for applications requiring data efficiency, computational practicality, and reliable feature interpretability essential factors in TB screening.

A TB screening system enhanced with deep learning (DL) not only delivers high diagnostic accuracy but has also been shown to reduce radiologists’ workload by approximately 15% [16]. In this study, we leverage the strengths of CNNs to effectively differentiate between active and latent TB on CXR images while addressing real-world clinical constraints and maintaining high interpretability.

Although deep learning has been shown to be promising for TB detection, previous studies have shown important limitations that interfere with clinical translation. First, the existing AI model mainly focuses on the dichotomy of TB and non-nuclear cases, which does not address clinically significant divisions between active and latent TB, which are prerequisites for implementing targeted interventions. Second, current approaches often prioritize diagnostic accuracy at the expense of analytical feasibility and a lack of visualization tools such as heat maps to locate pathological features, which radiologists need for clinical decision-making. Third, many models are learned in public datasets with heterogeneous annotation standards, limiting reliability in real-world environments where professional radiological expertise is essential.

In this study, we present a binary classification approach for distinguishing between active and inactive TB using a CNN model. The research process involved collecting CXR images from Cheonan Soonchunhyang Hospital, implementing and training the deep learning model and subsequently evaluating its performance.

The key contributions of this study are as follows:

(1) Development and implementation of an advanced deep learning approach to classify active and inactive TB instead of the TB/non-tuberculosis classification commonly covered in CXR images;
(2) Heatmap-based visualization of lesion locations to find pathological features;
(3) Professional data collection and annotation conducted by experts at Cheonan Soonchunhyang Hospital, not on public datasets;
(4) Application of the trained model in a computer-aided detection (CAD) system for future latent TB screening initiatives.

The remainder of this paper is organized as follows. Section 2 reviews the related work on TB classification, and Section 3 describes the proposed methodologies such as model and visualization. Section 4 describes experimental environments such as datasets, hyperparameters, and metrics, and Section 5 presents experimental results, discusses the findings, and compares them with recent relevant studies. Finally, Section 6 concludes the study and outlines future work.

## 2. Related Works

Recently, a variety of DL approaches have been proposed to address the challenge of distinguishing active TB from inactive TB or non-TB cases using CXR images. Several notable studies have explored different network architectures, pretraining strategies, and image enhancement techniques to improve classification performance. A summary of related works is provided below.

Choi et al. [17] conducted an active TB and inactive TB classification. The pretrained ResNet50 [18] on the ImageNet dataset was used as a baseline model. Then, the model performed another three pretraining phases (pneumonia vs. normal, pneumonia vs. active TB, and active TB vs. normal). After pretraining, the model was trained to classify active TB (3824 images) and inactive TB (2277 images). The model was validated on one internal and two external datasets and showed area under the curve (AUC) values of 98%, 81.5%, and 88.7%, respectively.

Lee et al. [19] implemented active TB and inactive TB classification. The EfficientNet [20] model was adopted as a baseline model and then fine-tuned to receive the output of a value between 0 and 1 for the probability of active TB. The model was trained and validated on a dataset consisting of a positive class (active TB or pretreatment (9836 images)) and a negative class (inactive TB or posttreatment (3327 images) and healthy (3182 images)). Then, the model was validated on two test sets and achieved AUC of 83% and 84% on test set 1 (active TB (80 images) and inactive TB (68 images)) and test set 2 (active TB (100 images) and inactive TB (100 images)), respectively.

Kazemzadeh et al. [21] developed active TB, inactive TB, and normal classification. Firstly, the Mask RCNN model [22] was used to crop lung region, and ResNet [18] was used to identify lesion areas. Then, EfficientNet [20] was used to define target class. The EfficientNet with Noisy Student semi-supervised learning was adopted as a baseline model and then fine-tuned to achieve the target classes. The model was trained and validated on the dataset (165,754 images). Then, the model was validated on the test set (1236 subjects, 17% with active TB) and achieved an AUC of 89%.

Munadi et al. [22] proposed a comparation of three image enhancements to classify positive (active TB) and negative (non-TB). Firstly, the input image is fed into image enhancement algorithms such as Unsharp Masking (UM) [23], High-Frequency Emphasis Filtering (HEF) [24], and Contrast Limited Adaptive Histogram Equalization (CLAHE) [25] to highlight the overall or local characteristics of the images. The baseline model, ResNet [18] and EfficientNet [20], are used to train on the enhanced images, and then fine-tuning is done to achieve the target classes. The models were tested on positive (active TB (336 images)) and negative (non-TB (326 images)). The Lightness Order Error (LOE) score was utilized to evaluate the performance of image enhancement algorithms of 837.89, 341.09, and 1852.1 for UM, HEF, and CLAHE, respectively. The ResNet and EfficientNet models with the UM algorithm achieved an AUC of 89.92% and 94.8%, respectively.

Pramana et al. [26] implemented active TB, non-TB, and healthy classification using Prototypical Networks for few-shot learning (FSL) [27]. The VGG16 [28], ResNet18 [18], and ResNet50 [18] models were adopted as feature extraction models, and then the models were tested with and without training methods with 3-way 20-shot, 3-way 10-shot, 3-way 5-shot, and 3-way 1-shot. The models were performed on a dataset of 11,200 images (train 80% and test 20%). For the without training method, VGG16, ResNet18, and ResNet50 achieved an accuracy of 71.77%, 74.80%, and 73.43%, respectively. For the with training method, VGG16, ResNet18, and ResNet50 achieved an accuracy of 33.33%, 98.93%, and 98.60%, respectively.

Tasci et al. [29] developed abnormal (active TB) and normal (non-TB) classification using voting-based ensemble learning. Firstly, the CLAHE [25] algorithm was utilized to enhance the images. Then, InceptionV3 [30] and Xception [31] models were adopted as baseline models, followed by fine-tuning to achieve the target classes. After that, Bayesian-optimization-based weighted voting ensemble learning with selected models according to their performance values was performed. The model was trained and validated on two datasets such as Montgomery (active TB 336 images and normal 326 images) and Shenzhen (active TB 287 images and normal 279 images) and achieved an accuracy of 97.5% and 97.6%, respectively.

Prasad et al. [32] investigated the classification of chest X-ray images into three categories: TB, Pneumonia, and Normal using Vision Transformer (ViT) [33] pretrained models, specifically ViTB-16 and ViTB-32, with transfer learning. Their study demonstrated that ViTB-16 outperformed ViTB-32 in TB identification, achieving a classification accuracy of 95.44%, a notable improvement over prior AI-based approach. Their work highlights the efficacy of self-attention mechanisms in medical image analysis and underscores the potential of transformer-based models to assist clinicians in diagnostic workflows.

The summary of each study is shown in Table 1.

## 3. Proposal Methods

Our study uses a deep learning method to classify active and inactive TB. We started by collecting, composing, and pre-processing data and then designing and implementing a deep learning model. In the following step, we will establish hardware and software environments for training and validation purposes. Finally, we tested our learned model and visualized the inferences in a heatmap.

### 3.1. Model Pipeline

**Figure 1:**
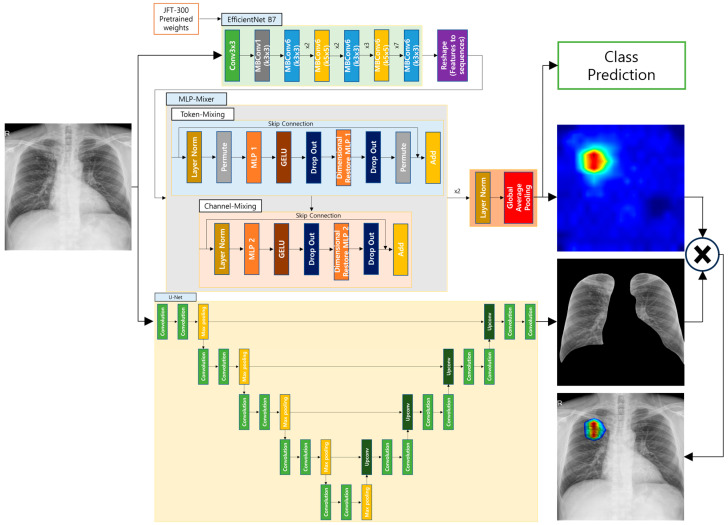
Overall proposed method pipeline.

The complete model pipeline is shown in Figure 1. Our first step is to train the proposed binary classification model and a semantic segmentation model [34] on the chest X-ray dataset. After training, the binary classification model produces class prediction and final convolutional layer feature maps, while the semantic segmentation model produces lung masks. Post-processed feature maps and mask data undergo visualization techniques to display visualization data.

### 3.2. Proposed Model

**Figure 2:**
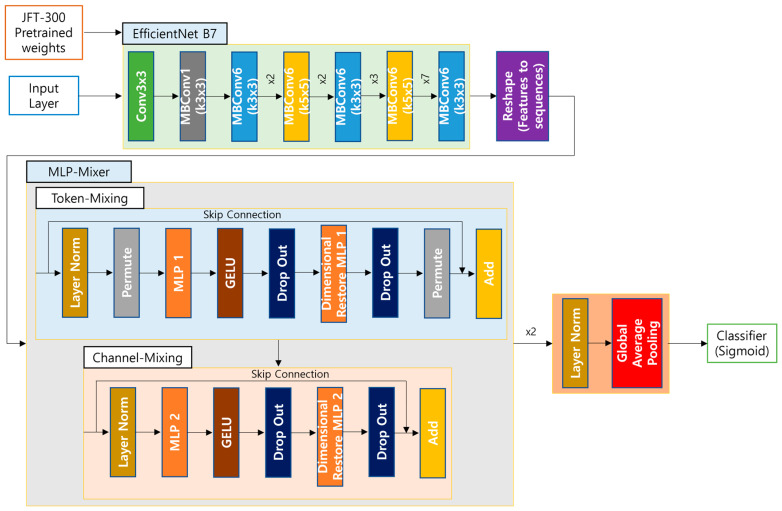
Overall proposed classification model.

To classify active and inactive TB, we used the EfficientNet [20] B7 model as the base model and then used a Multi-Layer Perceptron Mixer (MLP-Mixer) [35] as the decoder. To classify, the sigmoid function was utilized. Our proposed model is depicted in Figure 2.

In image classification tasks, EfficientNet performed well among CNN models with a top-1 accuracy of 84.4% trained on the ImageNet dataset. The EfficientNet model focuses on determining the optimal sizes of the receptive field width, depth, convolution layers, and input shape resolution. The EfficientNet B7 model consists of a stem block and seven MBConv blocks (Mobile Inverted Bottlenect Convolution).

We used the EfficientNet B7 model without the top layers as the backbone model and added new layers named MLP-Mixer because our dataset is small and highly diverse compared to the imageNet dataset. Before the MLP-Mixer layers, we used a reshape layer to sort the receptive field of the backbone layer into sequence. We linked up the norm-global average pooling layer and ultimately employed the sigmoid activation layer to obtain the probability of active TB within the range of the [0, 1] output. The predicted probability of inactive TB was computed in Equation (1):

p(inactive TB) = 1.0 − p(active TB)(1)

where p represents probability.

Our training utilized the transfer learning technique, which involves copying the learned weights of a model trained on RGB images. We employed pre-trained RGB image weights to train on CXR images because we believed they could capture more general features from a large dataset, which could be advantageous in identifying boundaries, shapes, textures, and more. Therefore, we adjusted our model using the weights from the JFT-300M dataset [36] that have already been trained using the Noisy Student [37] learning method.

Following the EfficientNet B7 backbone for feature extraction, we introduced two sequential MLP-Mixer layers to enhance cross-dimensional interaction and feature integration following the last activation layer. The MLP-Mixer architecture operates by translating input factors and explicitly modeling spatial (token mixing) and channel-wise (channel mixing) connections using dedicated MLP blocks.

In the first stage, token-mixing MLP processes flattened feature patches across spatial dimensions, aggregating global contextual information through a shared MLP applied independently to each channel. The next step is channel mixing MLP, which utilizes depth-wise MLP to fuse channel-specific features on specific tokens. In both mixing stages, Gaussian Error Linear Unit (GELU) activations and layer normalization are utilized, with skipped connections being used to conserve the gradient flow. The token- and channel-mixing blocks were duplicated twice as part of a hierarchical feature refinement cascade.

After the second Mixer layer, spatial features were collapsed using global average pooling, and the resultant embedding was projected to the target dimension via a linear layer followed by a sigmoid activation for classification. By leveraging the Mixer’s capacity to decouple spatial and channel-wise correlations, this design maintains parameter efficiency.

The MLP-Mixer adjusts the channel to have a dropout probability of 0.5 in the MLP layer. The hierarchy of mixing mechanisms in the CXR interpretation is in alignment with the hierarchy of features in the radial features. This hierarchical mixing mechanism can incorporate both local texture patterns in token mixing and disease-specific channel interactions in channel mixing.

In training, it has been proven that random zeroing some of the input elements can help regulate and prevent the co-adaptation of neurons (drop connect [38] and drop out [39] layers). A DropConnect layer, with a probability of 0.2 for setting input elements to zero, was implemented after the EfficientNet backbone layer. Furthermore, a dropout layer with a 0.5 probability was applied within the MLP layer. To optimize the training parameters on our data, we utilized the AdaBelief [40] optimizer. The learning rate was set to 4 × 10^−5^, weight decay to 1 × 10^−4^, global clipnormal to 1, rectify to true (for stabilizing the learning similar to Radam), and warmup proportion to 0.1 (for stabilizing the learning similar to the cosine annealing warmup restart learning scheduler).

To enhance the model’s ability to learn discriminative features from challenging cases, we adopted Focal Loss [41], a modified cross-entropy loss that prioritizes misclassified or ambiguous samples. The loss is defined as Equation (2):

(2) $$\mathrm{Binary}\;\mathrm{focal}\;\mathrm{cross}\;\mathrm{entropy}=-{\left(1-p_{t}\right)}^{\gamma }\cdot \log(p_{t})$$

where $p_{t}$ is the model’s predicted probability for the true class label. The focusing parameter γ (γ ≥ 0) controls how strongly the loss down-weights well-classified examples. When γ = 0, Focal Loss simplifies to standard cross-entropy. Higher values of γ progressively suppress the loss contribution of high-confidence predictions, compelling the model to focus on uncertain or borderline cases.

In our implementation, we set γ = 2.0 to prioritize difficult TB cases, such as lesions with overlapping radiographic features between active and inactive classes. The reason for setting γ to 2 is that it is the hyperparameter known to perform best in focal loss papers. This leads the model to pay more attention to subtle patterns that are important for clinical differentiation.

### 3.3. Semantic Segmentation Model

The semantic segmentation model in this study utilized the U-Net architecture to visualize the segmentation process. U-Net is a convolutional neural network originally developed for segmenting medical images, particularly effective even when the training dataset is relatively small. This makes it highly suitable for tasks like identifying cell boundaries or tumor regions.

Structurally, U-Net follows a symmetric design composed of a contracting path and an expanding path, connected through skip connections. The contracting part acts as the encoder, extracting hierarchical features by applying successive convolutional layers followed by max pooling. As this path progresses, the spatial resolution of the feature maps reduces while the number of channels increases, enabling the model to learn complex patterns.

The expanding path, functioning as the decoder, reconstructs the segmentation map by upsampling the features. During this reconstruction, the skip connections merge feature maps from the encoder with corresponding layers in the decoder, preserving spatial details that might otherwise be lost. This mechanism significantly improves the precision of the final segmentation output.

**Figure 3:**
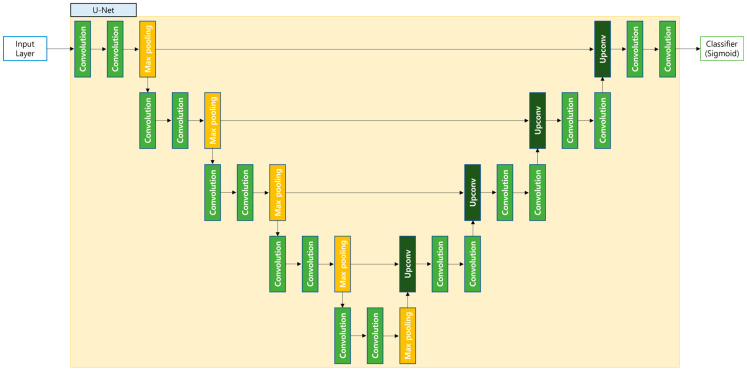
Overall structure of the semantic segmentation model based on U-Net.

One of the strengths of U-Net is its ability to integrate fine-grained spatial details with broader contextual information. This multi-scale feature integration results in accurate segmentation outputs. Moreover, with data augmentation techniques, U-Net generalizes well, even when trained on limited datasets. These qualities have contributed to its widespread adoption in medical image analysis and inspired many of its modern variants. A visual overview of this architecture is presented in Figure 3.

### 3.4. Mixed Precision

To enhance the computational efficiency of our training process while maintaining model performance, we implemented mixed precision training [42]. This approach strategically utilizes the strengths of both Float16 and Float32 numerical formats to optimize memory usage and accelerate computation. The key innovation of mixed precision lies in its ability to perform the majority of operations (forward pass, backward pass, and gradient computation) in Float16, which significantly reduces memory requirements and increases processing speed compared to conventional Float32 operations. However, to preserve numerical precision in critical operations that are sensitive to rounding errors, such as weight updates and loss computation, the system automatically maintains these specific calculations in Float32 precision.

The memory savings from mixed precision training provided us with greater flexibility in model experimentation. The reduced memory footprint enabled longer sequence lengths or more complex model architectures that might not have been feasible with full Float32 precision under the same hardware constraints. This aspect proved particularly valuable during our hyperparameter tuning phase, where the ability to rapidly test different configurations contributed significantly to our overall research efficiency.

### 3.5. Visualization

To improve the interpretability of model outputs, we utilized Gradient-weighted Class Activation Mapping (Grad-CAM) [43], which enables the identification of spatial regions that strongly influence the network’s predictions. This technique determines the relevance of each feature map by computing the average of gradients flowing from the target class score with respect to the last convolutional layer. These gradient-based weights are then used to produce a spatial attention map that emphasizes regions contributing most significantly to the model’s decision.

In this study, Grad-CAM was applied to the activation outputs from the final convolutional layer of our classifier to reveal areas potentially associated with pathological signs in CXR. To confine attention to lung-specific regions, the resulting saliency maps were multiplied element-wise with a binary mask generated via a lung segmentation model. This masking operation effectively suppressed irrelevant activations originating from non-pulmonary structures, such as mediastinum or external objects.

The resulting attention maps were visualized using the Jet colormap, where warmer colors indicate regions of higher importance. To reduce background noise and highlight diagnostically relevant regions, pixels below a defined threshold were filtered out. These enhanced attention maps were then superimposed onto the original radiographs through a weighted alpha blending process, where the opacity factor was adaptively tuned to maintain anatomical context while emphasizing areas of diagnostic significance.

**Figure 4:**
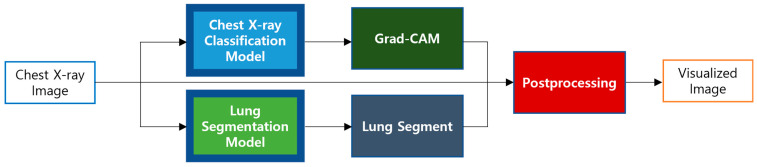
The visualization of the image heatmap overview.

Figure 4 presents an overview of this workflow, which integrates gradient-based saliency, anatomical masking, color mapping, and fusion steps. By combining Grad-CAM [43] with domain-specific constraints, the visualization becomes more clinically interpretable, reinforcing transparency and reliability in AI-assisted diagnostic tasks.

**Figure 5:**
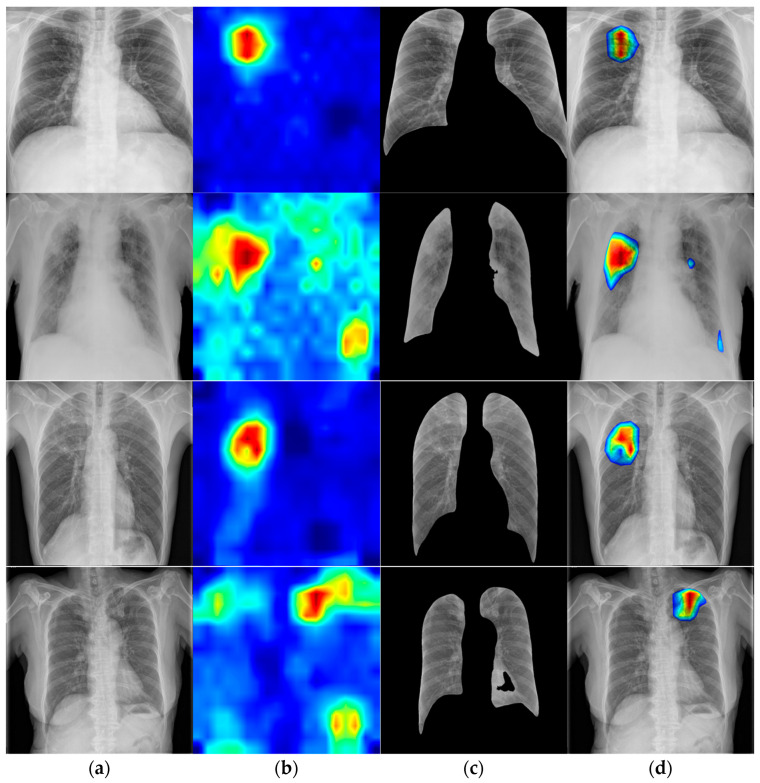
The inference visualization on SCH dataset: Grad-CAM analysis of TB cases. (**a**) CXR image: inactive TB (top two rows) and active TB (bottom two rows). (**b**) Model heatmap result: odd rows (*distinct*: sharp/localized) and even rows (*indistinct*: diffuse/scattered). (**c**) Lung segmentation result: anatomical masking for focused analysis. (**d**) Overlay result: post-processing the combined Grad-CAM activation map and lung segmentation mask.

In Figure 5, a comparative Grad-CAM visualization is presented for four patient cases, with the top two rows representing inactive TB and the bottom two rows corresponding to active TB. Each row illustrates a single patient case and displays four components in sequence: the original CXR image, the Grad-CAM-derived attention map highlighting the model’s focus, the segmented lung region generated by a dedicated segmentation model, and the final overlaid visualization combining the attention map with the anatomical mask.

The odd-numbered rows (first and third), associated with inactive TB cases, show tightly confined activation regions that align well with the anatomical boundaries of the lungs, indicating precise model attention to localized abnormalities. In contrast, the even-numbered rows (second and fourth), related to active TB, reveal broader and more diffuse activation patterns across multiple pulmonary zones. This suggests the model is responding to widespread or heterogeneous features characteristic of more advanced disease states.

These contrasting patterns underscore the model’s adaptive behavior in handling different disease presentations—effectively isolating specific features in localized cases while relying on distributed cues in more complex instances. The figure thus provides insight into both the interpretability of the model’s focus and its varying sensitivity to spatial feature distribution across TB types.

## 4. Experimental Environment

### 4.1. Dataset

The dataset includes 3828 CXR images that were collected and annotated by radiologists at Cheonan Soonchunhyang Hospital, and they have been ethically approved by the Institutional Review Board (IRB No. 2023-12-058-004). The DICOM files were transformed into 8-bit grayscale PNG format by removing any patient information.

The images were classified into two categories: active TB (1713 images) and inactive TB (2115 images). The dataset was divided into three mutually exclusive subsets: a training set (3064 images: 1371 active TB and 1693 inactive TB), a validation set (382 images: 171 active TB and 211 inactive TB), and a test set (382 images: 171 active TB and 211 inactive TB), as summarized in Table 2. Care was taken to ensure that no patient data overlapped across these subsets.

For dataset modality, we used digital radiography (DX), direct digital radiography (DR), and computed radiography (CR) and did not use computer tomography (CT). Only Posterior–Anterior (PA) and Anterior–Posterior (AP) position images were collected, and position images such as Decubitus and Lateral were excluded.

**Figure 6:**
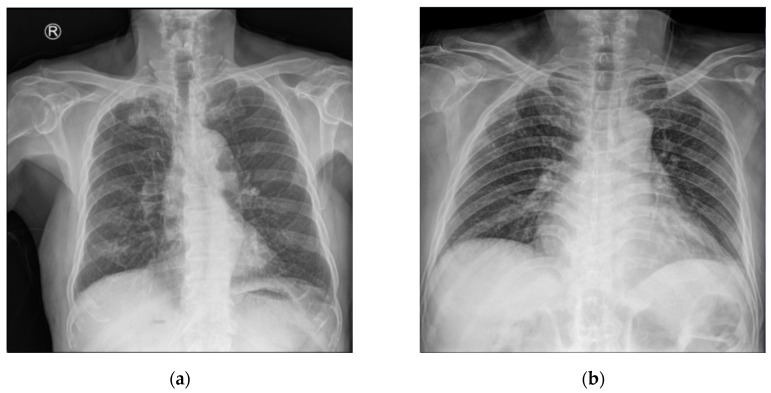

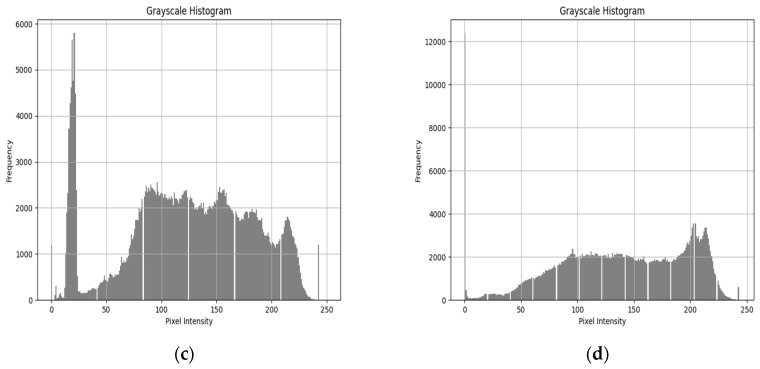
SCH dataset sample: (**a**) active TB; (**b**) inactive TB; (**c**) histogram of active TB; and (**d**) histogram of inactive TB.

Each CXR image is represented as a single-channel (grayscale) matrix, with pixel intensity values ranging from 0 to 255. Figure 6 presents representative examples and their corresponding intensity histograms: active TB cases (Figure 5a) typically show subtle parenchymal opacities, whereas inactive TB cases (Figure 5b) are characterized by fibrotic scars or calcified nodules. The histograms (Figure 5c,d) reveal substantial overlap in grayscale distributions between the two classes, indicating high inter-class similarity due to shared anatomical structures and low intra-class variability, with pathological changes localized mainly within the lung fields. These imaging characteristics pose significant challenges for both human clinicians and automated models as the discriminative features tend to be subtle and morphologically complex.

The dataset used for training the semantic segmentation model is an open dataset, the Shenzen Hospital Tuberculosis CXR Set [44] and the Montgomery County Tuberculosis CXR Set [45]. In total, 566 Shenzen CXR sets and 138 Montgomery CXR sets were used, and the ratio of the training set and test set was 8:2. This is shown in Table 3.

The proposed model’s multi-class performance was verified using an open dataset, the National Institutes of Health (NIH) dataset [46], as well. We utilized 10,000 datasets and divided the training and test sets 8:2 to utilize three classes: Normal, Pneumonia, and Pneumothorax. This is shown in Table 4.

### 4.2. Data Preprocsssing

Due to the rectangular nature of the original data, the width and height are not equal. In order to achieve a square shape, data were first cropped from the center. To accommodate the input format of the EfficientNet, the data were resized to (600, 600, and 3). Data were transformed into an input tensor with feature scaling from [0, 255] to [−1, +1]. The RandAugment method [47] was utilized to augment data with an N of 2 and an M of 28.

### 4.3. Environmental Setup

The model was trained on a hardware environment comprising an Intel Xeon Gold 6346 64-core CPU running at 3.10 GHz, four NVIDIA GeForce RTX 3090 GPUs, and 256 GB of RAM. The software environment included the HamoniKR 6.0 OS, CUDA 11.2, Anaconda 22.9, Python 3.9.13, TensorFlow 2.11.1, and Keras 2.11.0.

### 4.4. Hyperparameters

Table 5 summarizes our hyperparameter setting.

### 4.5. Evaluation Metrics

This study employed commonly used metrics to evaluate classification performance, namely, accuracy, sensitivity, and specificity. Accuracy reflects the overall proportion of correctly classified samples. Sensitivity measures how well the model identifies true positive cases, while specificity assesses its ability to correctly recognize true negatives. The exact definitions of these metrics are provided in Equations (3)–(5).

(3) $$Accuracy=\frac{TP+TN}{TP+TN+FP+FN}$$

(4) $$Sensitivity=\frac{TP}{TP+FN}$$

(5) $$Specificity=\frac{TN}{TN+FP}$$

where TP, FP, TN, and FN represent true positive, false positive, true negative, and false negative, respectively.

## 5. Experimental Results

### 5.1. Evaluation Result

Our model and hyperparameters were trained for 300 epochs; however, the best performance of validation was achieved at epoch 250. The best weights were selected based on the highest score of accuracy of the validation set. We achieved the best performance of validation with an accuracy of 94.5%, sensitivity of 95.3%, and specificity of 93.8%, as listed in Table 6.

Table 7 shows the confusion matrix of test set. Among 171 active TB, our model correctly predicted 157 images as active TB. Among 211 inactive TB, our model correctly predicted 205 images as inactive TB. Among 171 active TB, our model wrongly predicted 6 images as active TB. Among 211 inactive TB, our model wrongly predicted 14 images as inactive TB.

The best trained weight was inferenced on the test set (active TB (171 images) and inactive TB (211 images)) and achieved an accuracy of 96.3%, sensitivity of 95.9%, specificity of 97.2%, f1-score of 95.9%, and AUC of 98.6%, as shown in Table 8. Since sensitivity and specificity achieved a high and almost equal evaluation score, our model can be considered as a significant TB detection system with less false alarms.

The 95% Confidence Intervals (CIs) for each metric were (94.2–98.1%), (92.6–98.7%), (94–98.9%), (93.5–97.9%), and (97.3–99.5%), and the CIs were estimated using the bootstrap (10,000 random sampling with replacement) based percentile method (2.5–97.5%). Table 9 shows the Dice and IoU scores for the semantic segmentation task.

### 5.2. Comparison with Prior Work

Our model demonstrated competitive performance leveraging pre-trained weights, despite the limited dataset size. As summarized in Table 10, we compared the results of our model with the binary and multi-class classification benchmarks of prior work.

Compared to the same data characteristics (Choi et al. [17] and Lee et al. [19]), our AUC results achieved higher performance. Compared to Munadi et al. [22], we achieved almost the same performance. Moreover, compared to multi-class classification papers, our model outperformed Kazemzadeh et al. [21] by using the same EfficientNet as the baseline model. Moreover, compared to multi-class classification papers, our model outperformed Kazemzadeh et al. [21] and Prasad et al. [32] by using the same EfficientNet as the baseline model. When comparing the accuracy, our model showed slightly lower values than the binary classification study Pramana et al. [26] or the multiple classification study Tasci1 et al. [29].

### 5.3. Comparison with State-of-the-Art Methods

To further validate the efficacy of our proposed model, we conducted a comprehensive comparison with additional baseline architectures, including MobileNetV3 [48], DenseNet201 [49], InceptionV3 [30], EfficientNet B7 vanilla [20], ViT [33], ConvNeXt [50], and EfficientNet B7 integrated with five MLP layers [51].

**Figure 7:**
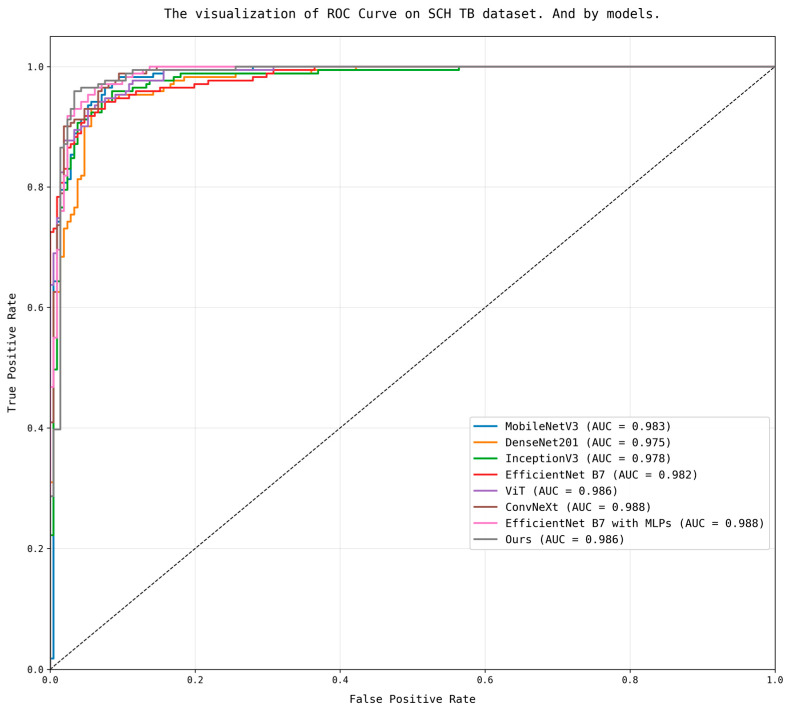
The visualization result of ROC curve by model (in Table 11).

First, when comparing AUC values using ROC curves, our model showed the third highest AUC value after EfficientNet B7 with MLPs and ConvNeXt, as shown in Figure 7, and was tied to ViT.

Secondly, when comparing the remaining performances, the results, ranked in descending order of performance, unlike AUC, demonstrated a clear hierarchy: our proposed model achieved superior performance, followed by EfficientNet B7 with MLPs, ConvNeXt, ViT, EfficientNet B7 vanilla, InceptionV3, DenseNet201, and MobileNetV3. Notably, our model, which also integrates MLP-based components, outperformed the EfficientNet B7 with MLPs, underscoring the superior design of our architectural refinements in capturing discriminative features critical for TB classification. ConvNeXt architecture, despite its modern design tailored for vision tasks, showed competitive but slightly lower accuracy, likely due to its sensitivity to limited training data. In the case of ViT, even though it is a model with performance that is replacing CNN, it shows similar performance to EfficientNet vanilla, perhaps because it is not a large dataset. Classical models like InceptionV3 and DenseNet201 exhibited moderate performance, while MobileNetV3 lagged significantly, possibly due to its lightweight structure sacrificing representational capacity for efficiency. This systematic evaluation underscores the potential of our approach combining CNNs and MLP-Mixer layers, as proposed in this work, to bridge the gap between computational efficiency and diagnostic accuracy in medical imaging. For the latency of the proposed model, the total latency was 27.21 s, and the per frame latency was 0.07 s. It is suitable for real-time Clinical Decision Support System (CDSS) models of less than 0.1 s, although not the best compared to other models. This contrasts with ViT and ConvNeXt, both of which exceeded 0.1 s. Table 11 shows the performance of each model.

In addition, when the multi-class performance of the model was measured using the NIH dataset, the performance was shown in Table 12. In the results, the performance of the proposed model was the best.

## 6. Conclusions

In this paper, we present a deep learning approach for the binary classification of active and inactive TB cases using CXR images. Our model leverages transfer learning, utilizing weights pre-trained on the JFT-300M dataset through the Noisy Student method. We specifically designed the model with an MLP-Mixer as the classification head, which played a crucial role in enhancing performance.

The model is trained on de-identified CXR images annotated by Cheonan Soonchunhyang Hospital. The collection of images was conducted according to the guidelines of the Declaration of Helsinki for research ethics and was approved by the Institutional Review Board of Soonchunhyang Hospital in Cheonan. In addition, it was de-identified through the deletion of patients’ privacy tags, and it complied with ethical standards by prohibiting use outside of study purposes.

On the validation set, it achieved an accuracy of 94.5%, sensitivity of 95.3%, and specificity of 93.8%. To further assess the model’s generalization ability, we evaluated it on an independent test set consisting of 382 CXR images, where it achieved an accuracy of 96.3%, sensitivity of 95.9%, and specificity of 96.6%. In comparison to recent studies, our model showed competitive results. These findings indicate that it has the potential to be an essential component in computer-aided diagnosis systems, resulting in faster clinical workflows and more effective early screening of latent TB. Since the average delay time per frame was 0.07 s, we also confirmed the possibility of using it for real-time CDSS with a delay of less than 0.1 s.

There were some limitations to this study. We compared prior studies on multi-class with our model, but this was not a direct comparison because the main training dataset was a type of binary classification. For this, we performed further verification with NIH, but it is true that further research is needed because it is not sufficient. In addition, when it comes to the comparison section of the SOTA model, the comparison group needs to be compared with various newly announced models because there are a few classic models.

In future work, we will strengthen the study of multi-classes and compare them with state-of-the-art SOTA models instead of classical models. Additionally, we plan to explore approaches that extract features at the patch level from segmented images, such as using deformable convolution. Furthermore, we aim to optimize computational efficiency while simultaneously improving performance on the model.

## Figures and Tables

**Table 1:** Comparison of models and reported performance in related works.

Paper	Class	Model	Task	AUC	Accuracy
Choi et al. [17]	Active TB, Inactive TB	ResNet50	Binary	0.887	-
Lee et al. [19]	Active TB, Inactive TB	EfficientNet	Binary	0.84	-
Kazemzadeh et al. [21]	Active TB, Inactive TB, Normal	EfficientNet	Multi-class	0.89	
Munadi et al. [22]	Active TB, Non-TB	EfficientNet	Binary	0.948	-
Pramana et al. [26]	Active TB, Non-TB, Healthy	ResNet18 (few-shot learning)	Multi-class	-	0.989
Tasci et al. [29]	Active TB, Normal	InceptionV3 and Xception (ensemble learning)	Binary	-	0.975
Prasad et al. [32]	TB, Pneumonia, Normal	ViT	Multi-class	0.954	

**Table 2:** Overview of SCH TB dataset.

	Training Set	Validation Set	Test Set	Total
Active TB ^1^	1371	171	171	1713
Inactive TB	1693	211	211	2115
Total	3064	382	382	3828

^1^ TB, tuberculosis.

**Table 3:** The dataset utilized in the segmentation task.

	Training Set	Test Set	Total
Lung X-ray data	562	142	704

**Table 4:** Overview of NIH CXR dataset.

	Training Set	Test Set	Total
Normal	2676	669	3345
Pneumonia	1114	278	1392
Pneumothorax	4210	1053	5263
Total	8000	2000	10,000

**Table 5:** Hyperparameter setup.

Parameter	Value
Input shape	600, 600, 3
Feature scaling	[−1, +1]
Data augmentation	RandAugment (N = 2, M = 28)
Regularization	Drop connect (0.5), drop out (0.5)
Optimizer	AdaBelief (Learning rate = 4 × 10^−5^, weight decay = 1 × 10^−4^, global clipnorm = 1, rectify = true, warmup proportion = 0.1)
Loss	Binary focal cross entropy (label smoothing = 0.1, γ = 2)
Classifier	Sigmoid
Batch size	32
Epoch	300

**Table 6:** Performance on the SCH CXR validation set at epoch 250.

	Accuracy	Sensitivity	Specificity
Validation set	94.5%	95.3%	93.8%

**Table 7:** Confusion matrix result on SCH CXR test set.

		Prediction	
		Positive	Negative
**Actual**	**Positive**	164	7
	**Negative**	7	204

**Table 8:** Model performance result on SCH CXR test set.

	Accuracy	Sensitivity	Specificity	F1-Score	AUC
Test set	96.3%	95.9%	96.6%	95.9%	98.6%
95% CI	(94.2–98.1%)	(92.6–98.7%)	(94–98.9%)	(93.5–97.9%)	(97.3–99.5%)

**Table 9:** Performance evaluation of the U-Net on the semantic segmentation task.

	Dice	IoU
Test set	94.4%	89.4%

**Table 10:** Quantitative evaluation results compared to previous works.

Paper	Class	Model	AUC	Accuracy
Choi et al. [17]	Active TB, Inactive TB	ResNet50	88.7%	
Lee et al. [19]	Active TB, Inactive TB	EfficientNet	84%	
Munadi et al. [22]	Active TB, Non-TB	EfficientNet	94.8%	
Kazemzadeh et al. [21]	Active TB, Inactive TB, Normal	EfficientNet	89%	
Prasad et al. [32]	TB, Pneumonia, Normal	ViT	95.4%	
Pramana et al. [26]	Active TB, Non-TB, Healthy	ResNet18 (few-shot learning)		98.9%
Tasci1 et al. [29]	Active TB, Normal	InceptionV3 and Xception (ensemble learning)		97.5%
Ours	Active TB, Inactive TB	EfficientNet	98.6%	96.3%

**Table 11:** Quantitative performance comparison results on SCH TB dataset.

Model	Accuracy	Sensitivity	Specificity	F1-Score	Per-Frame Latency
MobileNetV3 [48]	90.5%	82.4%	97.1%	88.3%	0.04 s
DenseNet201 [49]	92.6%	90%	94.7%	91.6%	0.06 s
InceptionV3 [30]	92.9%	94.7%	91.4%	92.3%	0.05 s
EfficientNet B7 [20]	93.4%	90.6%	95.7%	92.5%	0.07 s
ViT [33]	93.4%	92.98%	93.84%	92.71%	0.15 s
ConvNeXt [50]	94.2%	92.9%	95.2%	93.5%	0.14 s
EfficientNet B7 with MLPs [51]	94.7%	91.8%	97.1%	94%	0.07 s
Ours	96.3%	95.9%	96.6%	95.9%	0.07 s

**Table 12:** Multi-class performance comparison result on NIH dataset.

Model	Accuracy	Sensitivity	Specificity
VGG 19 [28]	84.25%	76.36%	88.18%
DenseNet201 [49]	84.37%	76.56%	88.28%
EfficientNet B7 [20]	84.76%	77.14%	88.57%
EfficientNet B7 with multi-GAP [13]	85.15%	77.73%	88.86%
ConvNeXt [50]	85.38%	78.07%	89.03%
Ours	85.45%	78.17%	89.09%

## Data Availability

No new data were created in this study. Data sharing is not applicable.
